# Rare myeloid sarcoma with *KMT2A* (*MLL*)-*ELL* fusion presenting as a vaginal wall mass

**DOI:** 10.1186/s13000-019-0804-6

**Published:** 2019-03-28

**Authors:** Haiyan Bao, Juehua Gao, Yi-Hua Chen, Jessica K. Altman, Olga Frankfurt, Amanda L. Wilson, Madina Sukhanova, Qing Chen, Xinyan Lu

**Affiliations:** 1 Department of Pathology, Northwestern University Feinberg School of Medicine, 303 East Chicago Avenue, ward 3-140, Chicago, IL 60611 USA; 2 Department of Hematology, The first affiliated hospital of Soochow Univervisty, Suzhou, Jiangsu, China; 3 Department of Hematology-Oncology, Northwestern University Feinberg School of Medicine, Chicago, IL USA; 4 Department of Pathology, Aurora Medical Center, Pathology, Kenosha, WI USA

**Keywords:** Myeloid sarcoma, Histiocytic sarcoma, *KMT2A-ELL* fusion, Molecular, Cytogenetics, Copy number aberrations, SNP microarray, OncoScan

## Abstract

**Backgroud:**

Myeloid sarcoma (MS) is a rare neoplasm of immature myeloid precursors that form tumor mass outside the bone marrow. The diagnosis of de novo MS can be challenging, particularly in patients with no prior history of hematologic malignancies or when MS involves unusual anatomic sites.

**Case presentation:**

The patient was a 53-year-old woman with a history of uterine fibroids and vaginal bleeding for many years who presented with a vaginal wall mass. The tumor had histologic and phenotypic features of histiocytic sarcoma, however, overlapping with a possible extramedullary MS. Using a comprehensive genomic profiling, we were able to identify recurrent chromosomal aberrations associated with MS including a rare *KMT2A*-*ELL* fusion, losses of chromosomes 1p, 9, 10, 15, 18, and gain of chromosome 1q and mutations in *FLT3* and *PTPN11*, and achived the final diagnosis of a de novo MS. The patient received standard treatment for acute myeloid leukemia regimen with stem cell transplantation and achieved complete remission.

**Conclusion:**

Our case illustrates the clinical utility of comprehensive genomic profiling in assisting the diagnosis or differential diagnosis of challenging MS or histiocytic sarcoma cases, and in providing important information in tumor biology for appropriate clinical management.

## Backgroud

Myeloid sarcoma (MS) is a rare neoplasm of immature myeloid precursors that form tumor mass outside the bone marrow [1]. It can occur as de novo tumor, recurrent acute myeloid leukemia (AML), or blastic transformation of myelodysplastic syndrome (MDS), or myeloproliferative neoplasm [2]. Skin, lymph nodes, gastrointestinal tract and soft tissue are the most common sites for MS involvement. The diagnosis of de novo MS can be challenging, particularly in patients with no prior history of hematologic malignancies or when MS involves unusual anatomic sites [3]. In recent years, with better understanding of the genomic profiling of myeloid neoplasms (MN), cytogenetic and molecular technologies have been increasingly utilized as important ancillary studies in the diagnosis of difficult MS cases [4]. Here we describe a case of de novo MS occurring in an unusual location as a solitary vaginal wall mass, with overlapping histologic and phenotypic features with histiocytic sarcoma (HS).

### Case presentation

The patient was a 53-year-old woman with a history of uterine fibroids and vaginal bleeding for many years who presented with a vaginal wall mass. She underwent total laparoscopic hysterectomy and resection of vaginal mass. Intraoperatively, it was noted that she had fibroids, and the bilateral ovaries and fallopian tubes were normal. There was a 5 × 8 cm mass arising from the right sidewall of vagina.

## Materials and methods

### Immunohistochemical analysis

Immunohistochemical staining was performed on 4 μm formalin-fixed and paraffin-embedded (FFPE) tissue sections using VENTANA BenchMark system (Roche, Indianapolis, IN) following standard laboratory procedures. The following antibodies were used in the diagnostic work-up: anti-CD45, CD43, Lysozyme, CD4, CD68, CD163, CD34, CD117, myeloperoxidase (MPO), CD3, CD20, CD30, ALK-1, CD21, S-100, HMB-45/Mart 1, SMA, desmin, synaptophysin, and PAX-8 (Dako, Carpinteria, CA).

### FISH and OncoScan analysis

Fluorescence in situ hybridization (FISH) analysis was performed using Vysis® LSI® (Abbott Park, IL) dual color, break apart probes for detection of rearrangements of *KMT2A (MLL)* and *CBFB,* and dual color, dual fusion probe set for detection of t(8;21)*/RUNX1T1-RUNX1* fusion. FISH analysis was performed on 4 μm FFPE slides to detect known recurrent cytogenetic aberrations associated with MS, following standard laboratory procedures. A total of 200 cells were counted by two technologists independently.

Genomic DNA was extracted from FFPE specimens with QIagen Dneasy Blood & Tissue Kit (Qiagen Inc. Valencia, CA), according to the manufacturer’s instructions. Single nucleotide polymorphism (SNP) microarray testing was performed using the Affymetric OncoScan™ arrays (Affymetrix/Thermo Fisher Scientific, Santa Clara, CA) following the manufactrer’s procedure.

### Molecular profiling

Compherensive genomic profiling test with the FoundationOne Heme panel of genes was performed by Foundation Medicine, Inc. (Cambridge, MA) based on published methods. FoundationOne Heme is validated to detect genomic alterations in more than 400 cancer-related genes. FoundationOne Heme employs RNA sequencing across more than 250 genes to capture a broad range of gene fusions, common drivers of hematologic malignancies, and sarcomas.

## Results

**Fig. 1:**
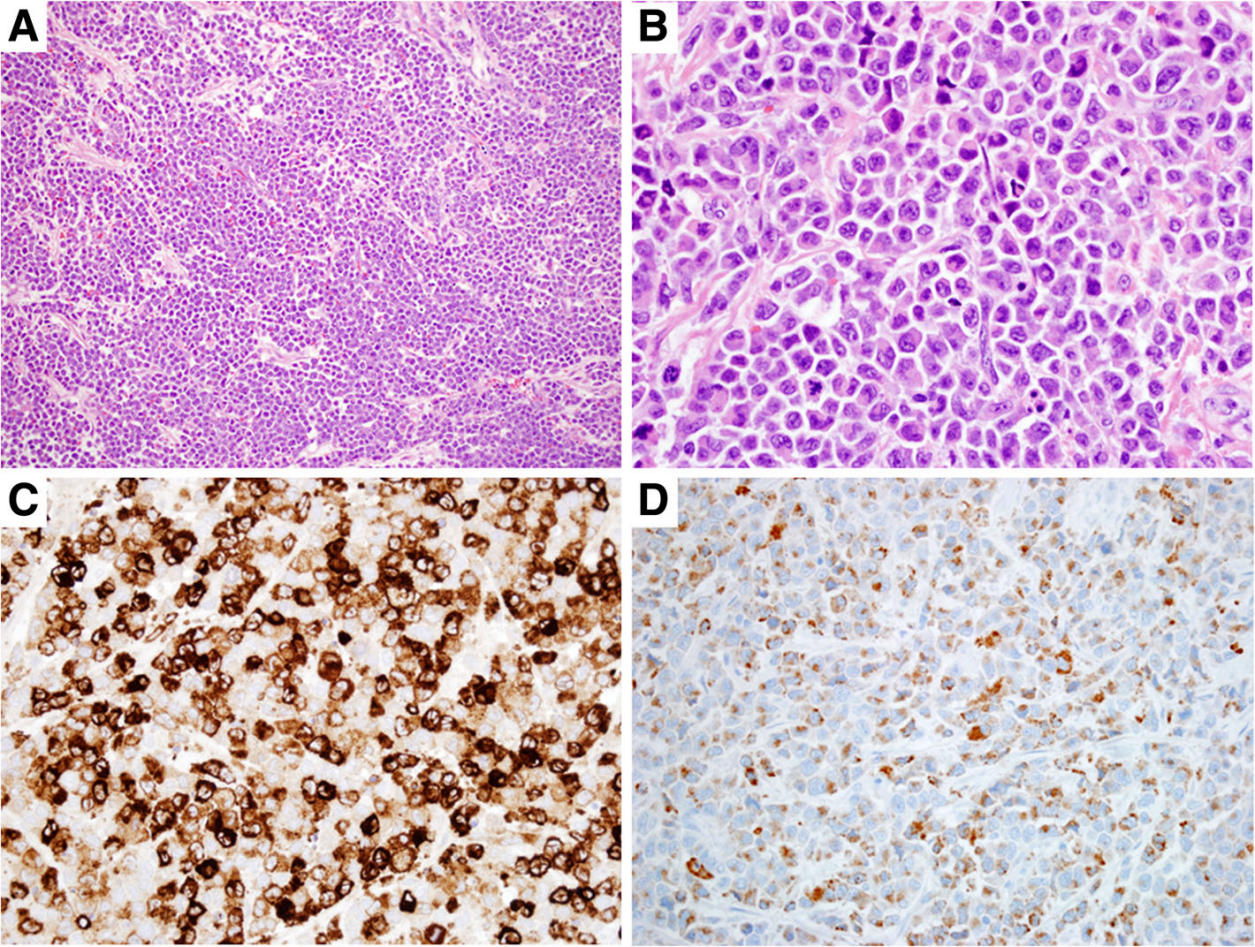
Myeloid sarcoma with initial presentation as a vaginal wall mass. Histologic sections reveal extensive infiltrate by malignant cells that are large with irregular folded nuclear contours, open chromatin, variably prominent nucleoli and abundant cytoplasm (**a**. HEx200, **b**. HE × 400). The neoplastic cells are variably positive for CD163 and weakly positive for CD68 (**c**. CD163 × 400, **d**. CD68 × 400)

Histological sections of the vaginal mass showed extensive infiltrate by malignant cells that were large in size with irregular/folded and sometimes lobulated nuclear contours, open chromatin, variably prominent nucleoli and abundant cytoplasm. Mitosis was brisk, and surface erosion and focal necrosis were present (Fig. 1). Immunohistochemical studies showed that the neoplastic cells were positive for CD45, CD43, Lysozyme, CD4, CD68 (weak), CD163 (variable), CD56, and vimentin, and negative for CD34, CD117, myeloperoxidase, CD3, CD20, CD30, ALK-1, CD21, S-100, HMB-45/Mart 1, SMA, desmin, synaptophysin, and PAX-8. In situ hybridization for EBER (Epstein-Barr virus-encoded RNA) was negative. A bone marrow biopsy was performed and showed no evidence of AML or other myeloid malignancies. Although histological findings favored a MS with monocytic differentiation, the possibility of HS could not be completely ruled out given the morphologic and immunophenotypic overlap of these two neoplasms.

**Fig. 2:**
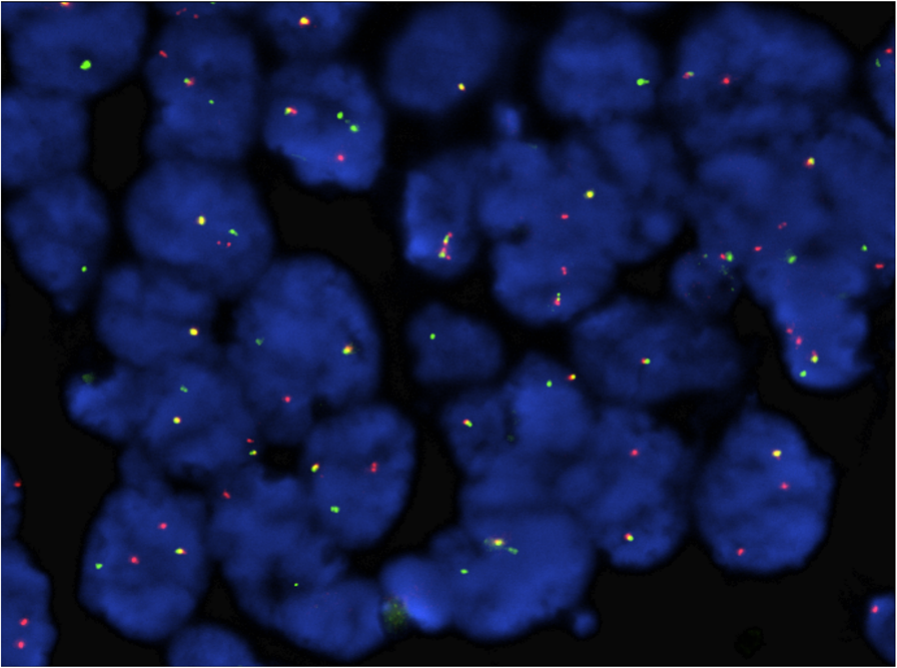
Fluorescence in situ hybridization (FISH) for *KMT2A (MLL*) rearrangement. FISH analysis using *KMT2*A dual color break apart probe was performed on formalin fixed paraffin embedded tissue section. The tumor cells demonstrated separation of the signals indicating *KMT2A* gene rearrangement in 179 of 200 cells analyzed

**Fig. 3:**
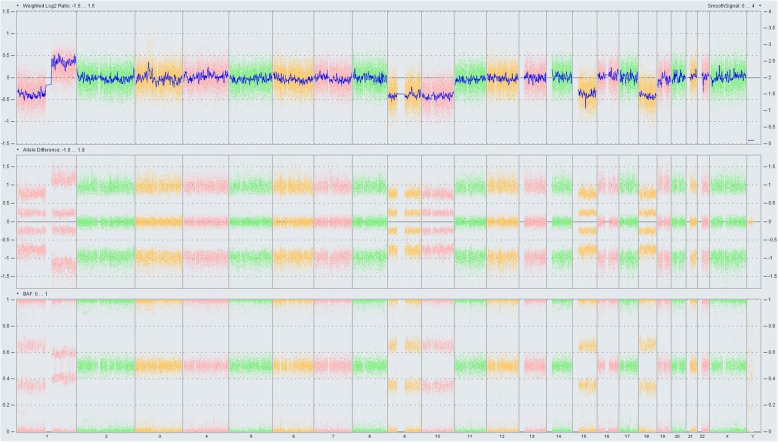
Global chromosome copy number changes by Oncoscan analysis. Oncoscan analysis revealed copy number changes involving multiple chromosomes including losses of 1p, 9, 10, 15, 18 and, gain of 1q

FISH analysis on 4 μm FFPE slides identified a *KMT2A* (*MLL*) gene rearrangement, a recurrent genetic abnormality in MS, in 89.5% of cells examined in this case (Fig. 2). FISH analysis was negative for *CBFB* rearrangement or *RUNX1T1-RUNX1* fusion. Due to limited material available, conventional cytogenetics could not be performed. OncoScan SNP microarray analysis revealed losses of chromosomes 9, 10, 15, and 18, and loss of the short arm and gain of the long arm of chromosome 1 (Fig. 3). Additional next generation sequencing (NGS) analysis performed by Foundation Medicine revealed multiple genomic alterations including *FLT3* S451F, *CHEK2* T367 fs*15, *PTPN11* A72V, *RAD21* N462 fs*1, and most importantly, an *KMT2A-ELL* fusion. The neoplastic cells showed low Tumor Mutation Burden (3 Muts/Mb) and the Microsatellite status was stable. The identification of multiple genetic/molecular abberations typically seen in myeloid neoplasms by integrated molecular and genomic profiles strongly supported the diagnosis of MS.

The patient received standard 7 + 3 (idarubicin and cytarabine) induction chemotherapy for AML. She tolerated the treatment well and subsequent PET CT showed no evidence of disease. She received 2 cycles of consolidation therapy followed by a myeloablative allogeneic matched unrelated donor (MUD) hematopoietic stem cell transplant (HSCT). The patient has been in complete remission since then.

## Discussion and conclusions

The diagnosis of MS requires demonstration of myeloid blasts in a mass forming lesion involving an extra medullary site. Based on morphologic grounds alone, the differential diagnosis often remains broad, including lymphoma, undifferentiated carcinoma, and small round blue cell tumor such as neuroblastoma, rhabdomyosarcoma, Ewing sarcoma/PNET, or medulloblastoma. Most of these entities are not difficult for differential diagnosis using appropriate immunohistochemical stains or molecular cytogenetics testing. The neoplastic cells in MS often express precursor markers CD34 and/or CD117 together with granulocytic or monocytic markers such as MPO, CD13, CD33, CD68, and lysozyme. Different studies reported variable expression frequencies of MS for MPO (63–92%), CD13 (48%), CD33 (48–52%), CD68 (35–61%), and lysozyme (26–100%) [2, 5–7]. MS with monocytic differentiation can be particularly challenging to differentiate from histocytic sarcoma (HS) as both can express one or more monocytic antigens, such as CD68, CD163 and lysozyme. Although HS may exhibit higher degree of cytologic atypia with more pleomorphic or anaplastic cytomorphology and less blastic appearance, the morphologic features are not distinct enough to differentiate MS from HS. In our case, the neoplastic cells exhibited overt atypia with frequent cells showing anaplastic features. Therefore HS was considered in the initial differential diagnosis. Since HS has the phenotypic characteristics of tissue histiocytes, which are derived from monocyte-macrophage lineage, the neoplastic histiocytes express CD4, CD14, CD163, CD68, and lysozyme, similar to the blood and bone marrow monocytes [8]. The presence of myeloid precursor markers such as CD34, CD117, and MPO, if present, would support a diagnosis MS. However, these markers are absent in our case. Aberrant expression of CD56 would also favor MS, but rare cases of HS can also express CD56 [9]. Therefore, our case represents an example of diagnostic dilemma between MS and HS based on morphological and immunhistochemical features.

At genetic level, there are notable differences between MS and HS. Cytogenetic aberrations are common in MS. Miyoshi et al. reported an abnormal karyotype in 73.2% (41/56) of MS patients, and many cases had a complex karyotype as indicated by the Oncoscan SNP microarray analysis [2], although no unique pattern for chromosomal abnormalities has been reported for MS. Previous studies have suggested that MS is more likely to be associated with certain translocations such as *CBF* family genes or *KMT2A (MLL)* rearrangements [10]. AML with *KMT2A* rearrangements involves a number of translocations of the *KMT2A* gene with different partners. In this case, FISH was positive for a translocation involving the *KMT2A* gene and the next generation sequencing testing further defined the rare t(11;19)/*KMT2A*-*ELL* fusion. The t(11;19)(q23.3;p13.1) translocation, involving the *KMT2A* and *ELL* genes, is a recurrent abnormality in AML, acute lymphoblastic leukemia and mixed phenotype acute leukemia. A recent study reported that among all known *KMT2A* translocation partners, *KMT2A*-*ELL* fusions are present in 12% of adult AML, 7% of pediatric AML and 15% of infant AML [11]. The exact role of *KMT2A*-*ELL* in AML pathogenesis is unclear, but animal studies showed that the fusion protein provided an enhancing effect on the proliferative potential of hematopoietic progenitors [12]. Additionally, MS shares the same mutation spectrum as AML, frequently involving RAS pathway, activated signals, DNA methylation, cohesins, splicing, transcription factors, chromatin modification and other myeloid neoplasm-related genes. *NPM1*, *NRAS*, and *DNMT3A* are found to be most frequently mutated in AML [13]. The other affected genes include *TET2*, *FLT3*-ITD/TKD, *PTPN11*, *IDH2*, *CSF3R*, *RUNX1*, *GATA2*, and *ASXL1*. In our case, in addition to the *KMT2A*-*ELL* fusion, mutations associated with myeloid neoplasms, such as *FLT3* and *PTPN11*, were also detected, indicating cooperative roles of these genetic alterations in the development of this MS case. *PTPN11* is a negative regulator of the RAS/MAPK pathway. Mutations in *PTPN11* result in activated MAPK signaling. Rare cases of HS with *PTPN11* mutations have been reported and showed response to the MEK inhibitor trametinib that blocks the RAS/MAPK pathway [14]. The OncoScan SNP microarray analysis also revealed muliptle chromosomal gains and losses along with the *KMT2A*/*ELL* fusion, inidicative of the presence of complex aberrations that are known to be associated with meyolid neoplasms, e.g. MS, and further support the final diagnosis of MS in this patient.

There are limited reports of chromosomal analysis on HS. The t(14;18) and trisomy 8 have been reported in rare cases of HS arising in the setting of previously diagnosed follicular lymphoma or chronic myelomonocytic leukemia, probably from a common neoplastic precursor [15]. On the other hand, recurrent gene mutations in the Ras/Raf/MEK/ERK signaling pathway have been detected in histiocytic neoplasms [16]. For example, *BRAF* V600E mutation is well described in histiocytic neoplasms such as langhans cell histiocyotosis, Erdheim-Chester disease, and histiocytic sarcoma. The presence of *BRAF* mutation would support the diagnosis of histiocytic neoplasm as *BRAF* mutations are generally considered not present in acute monocytic/monoblastic leukemia [17]. The present case showed negativity of *BRAF* mutation. In addition to *BRAF*, activating *ARAF*, *RAS*, and *MAP2K1* mutations, as well as activating fusions in *BRAF*, *ALK*, and *NTRK1* have been reported in histiocytic neoplasms [18].

Although there is significant overlap in morphology and immunophenotype between MS with monocytic differentiation and HS, the clinical management is very different. MS is a presentation of AML and should be treated as such. There is no consensus on the standard treatment for HS, and the patients are usually treated with combined chemotherapy. Therefore, it is very important to make the accurate diagnosis so that the patients can receive appropriate therapy.

We report a rare case of MS with unusual clinical presentation and morphologic features overlapping with HS. A comprehensive genomic approach allowed us to identify several cytogenetic and molecular alterations characteristic of MNs. The combination of positivity of myeloid associated mutations and negativity of common HS related mutations further confirmed the present case is a myeloid sarcoma. Our case illustrates the importance of genomic studies in establishing the correct diagnosis in morphologically challenging cases. Furthermore, comprehensive genomic profiling may identify recurrent alterations that are suitable for targeted therapy. This case further confirms the consensus that although MS and HS share overlapping histologic and phenotypic features, they are genetically distinct entities.

## Abbreviations

AML: Acute myeloid leukemia
HS: Histiocytic Sarcoma
MDS: Myelodysplastic syndrome
MN: Myeloid neoplasm
MS: Myeloid Sarcoma
SNP microarray: Single nucleotide polymorphism microarray
